# Supplementary material for: The association between the triglyceride-glucose index with all-cause and cardiovascular mortality within the infertility population
Source: PLoS One. 2025 May 7;20(5):e0320526. doi: 10.1371/journal.pone.0320526 (PMC12057929; doi:10.1371/journal.pone.0320526)
Supplement: Attachment — Submitted filename: Response to reviewers.docx [file pone.0320526.s002.docx]

**Response to reviewers:**

This interesting study highlights the relationship between the TyGI and the all mortality in the infertile women. there are few points need to be clarify
1. Introduction: A paragraph explaining why infertile women are at risk for cardiovascular events. Add the rationale of the study

Reply: Thank you for your kind reminder, and we have revised the first paragraph in the Introduction section, making it clearer for reviewers and readers about the rationale for conducting this research.

2. Methods: why the authors collected the data up to 2018. The statistic analysis requires more details as the results showed findings with specific statistical tests.

Reply: We only collected data up to 2018 as self-reported infertility only occured in the three cycles of 2013-2018 (2013-14, 2015-16, 2017-18). Other cycles did not include this question in the interview.

3. Results: The flowchart (figure 1) is without legend and the tables without footnotes explaining the table thoroughly.

Reply: We have Added the legend in figure 1 and footnotes in the tables.

4. References: many typing errors

Reply: We collated the references with Endnote and applied the “Vancouver” style.

5. The overall abbreviations require major revision

Reply: The abbreviations have been corrected.
